# Impact of Serum Phosphate, Potassium and Other Electrolyte Levels on Sudden Cardiac Death and Cardiovascular Mortality in Haemodialysis and Peritoneal Dialysis: A Systematic Review and Meta-Analysis

**DOI:** 10.3390/biomedicines14030605

**Published:** 2026-03-09

**Authors:** Beata Franczyk, Jacek Rysz, Anna Gluba-Sagr

**Affiliations:** 1 Department of Nephrocardiology, Medical University of Lodz, Zeromskiego 113, 90-549 Lodz, Poland; beata.franczyk-skora@umed.lodz.pl; 2 Department of Nephrology, Hypertension and Family Medicine, Medical University of Lodz, Zeromskiego 113, 90-549 Lodz, Poland; jacek.rysz@umed.lodz.pl

**Keywords:** cardiovascular mortality, haemodialysis, peritoneal dialysis, serum electrolyte level disturbances

## Abstract

**Background**: Dialysis patients have a very high burden of cardiovascular mortality, yet the contribution of specific serum electrolytes to sudden cardiac death (SCD) and cardiovascular death across haemodialysis (HD) and peritoneal dialysis (PD) remains uncertain. **Methods**: We conducted a PROSPERO-registered systematic review and meta-analysis (2010–2025) of cohort studies reporting adjusted hazard ratios (HRs) for the association between baseline or time-averaged serum electrolytes and cardiovascular mortality or SCD in adult maintenance HD and/or PD. Random-effects models with modality-specific and pooled analyses were applied. **Results**: Thirty-five cohorts (over 200,000 patients) met inclusion criteria. Across modalities, categorical analyses showed that high phosphate and low magnesium were consistently associated with approximately 2-fold higher cardiovascular mortality, while extreme potassium categories conferred similar excess risk, driven largely by PD. In HD, hypomagnesaemia and hyperphosphataemia were each associated with around 2-fold higher risk, and lower continuous sodium levels were linearly related to higher cardiovascular mortality. In PD, severe potassium abnormalities, hypomagnesaemia and high phosphate categories were strongly associated with cardiovascular death, and a lower Na/Cl ratio identified patients at particularly high risk. Heterogeneity was generally modest for categorical magnesium and phosphate, but substantial for some potassium and continuous-exposure models. Sensitivity analyses confirmed the robustness of key findings. **Conclusions**: Across HD and PD, abnormalities in phosphate, magnesium, potassium and sodium are strong and largely consistent markers of cardiovascular mortality, and likely SCD, with important modality-specific patterns. These data support intensified, modality-tailored management of electrolyte profiles as a central component of cardiovascular and SCD risk reduction in dialysis.

## 1. Introduction

Patients with end-stage kidney disease (ESKD) treated with maintenance haemodialysis (HD) or peritoneal dialysis (PD) experience an exceptionally high burden of cardiovascular morbidity and mortality compared with the general population [1]. Sudden cardiac death (SCD), typically defined as fatal cardiac event occurring unexpectedly and within one short interval after symptom onset, is particularly prominent and is responsible for roughly one in four deaths or more in haemodialysis cohorts [2,3,4,5]. Non-sudden cardiovascular deaths, including fatal arrhythmias, myocardial infarction, and progressive heart failure, also remain disproportionately common among dialysis patients [6]. These outcomes reflect a complex interaction between structural heart disease, autonomic dysfunction, chronic myocardial injury, and acute physiological stressors related to the dialysis procedure itself [7,8,9]. Electrolyte and mineral disturbances are central to this cardiovascular vulnerability. HD and PD patients are exposed to marked fluctuations in serum electrolytes due to impaired renal clearance, interdialytic accumulation, variable dietary intake, and rapid intradialytic shifts [10]. Such changes influence myocardial excitability, vascular tone, repolarization stability, and hemodynamic resilience, and may therefore contribute to both SCD and broader cardiovascular mortality [8,11]. Among these factors, serum magnesium, sodium, chloride, and calcium have emerged as potentially modifiable determinants of cardiovascular risk [12]. Low serum magnesium has consistently been associated with higher cardiovascular mortality and arrhythmic risk in several dialysis cohorts [13,14,15]. Observational studies have reported that hypomagnesaemia is common in HD, may predispose to ventricular arrhythmias, and is independently associated with fatal cardiovascular events [13,14,15,16]. Disturbances in sodium balance also influence cardiovascular outcomes. Hyponatraemia predicts higher cardiovascular mortality in both HD and PD patients and is strongly associated with neurohormonal activation, inflammation, and myocardial stress [17,18,19,20]. In addition, rapid intra-dialytic sodium shifts may promote myocardial stunning, intradialytic hypotension, and subsequent cardiovascular events [21].

Observational studies have linked both nadir and peak serum potassium concentrations to rhythm disorders and SCD. Numerous cohorts suggest that extremes of potassium, both low and high, contribute to electrical instability, and steep serum-to-dialysate potassium gradients have been associated with cardiac arrest [22,23].

Serum chloride, although less frequently studied, has shown independent prognostic value in dialysis populations. Lower chloride concentrations have been associated with higher cardiovascular mortality in HD [24], while in PD patients, higher baseline chloride has been linked to increased cardiovascular risk, suggesting potential modality-specific or context-dependent effects [25]. Calcium balance represents another critical determinant: elevated serum calcium promotes vascular and myocardial calcification, arterial stiffness, and left ventricular hypertrophy, all of which increase cardiovascular mortality risk [26,27,28]. Moreover, large gradients between serum and dialysate calcium concentrations may precipitate acute hemodynamic instability and arrhythmia during dialysis [29].

Despite strong biological plausibility and expanding observational evidence, findings across studies remain inconsistent due to differences in electrolyte cut-offs, exposure definitions, dialysis modality, outcome definitions (SCD vs. cardiovascular mortality), and confounder adjustment. Existing reviews have primarily focused on hyponatraemia or broader mineral metabolism (calcium–phosphate–PTH axis), with few analyses synthesizing risk estimates across multiple electrolytes while distinguishing between HD and PD populations. Only a limited number of systematic meta-analyses have stratified by modality or quantified pooled hazard ratios across adult populations [22,30].

To address these gaps, we performed a systematic review and meta-analysis to evaluate the association between serum phosphate, potassium, magnesium, chloride, and calcium levels, related indices (including Na/Cl ratio and Ca × P) and the risk of SCD and cardiovascular mortality among adults receiving HD or PD. By stratifying analyses by dialysis modality and standardizing effect estimates across studies, we aimed to clarify the contribution of these electrolyte abnormalities to fatal cardiovascular events and identify potential targets for improved risk management in the dialysis population.

## 2. Materials and Methods

### 2.1. Research Registration

This systematic review was carried out in accordance with PRISMA 2020 statement and guidelines from the Cochrane Handbook. The protocol was prospectively registered in PROSPERO (registration number: CRD420251239539). No amendments were made after registration.

### 2.2. Literature Search Strategy

We conducted a comprehensive and structured search of PubMed, the Cochrane Central Register of Controlled Trials (CENTRAL), and Google Scholar to identify all relevant studies examining the associations between serum electrolyte levels (magnesium, potassium, calcium, sodium, and phosphate) and the risk of cardiovascular mortality or SCD in adult dialysis patients. The search covered publications from January 2010 to October 2025. and was last executed on: PubMed (1 October 2025), Cochrane CENTRAL (2 October 2025) and Google Scholar (5 October 2025). Reference lists of included studies and relevant reviews were screened on 20 October 2025.

In each database, we combined controlled vocabulary terms and free-text keywords related to SCD and cardiovascular mortality, serum electrolytes, and renal replacement therapy (HD, PD, and related terms). No language or publication-type restrictions were applied at the initial search stage; however, for quantitative synthesis we included only full-text articles available in English that reported adjusted hazard ratios (aHRs) with 95% confidence intervals (CIs) for cardiovascular mortality and/or SCD. In Google Scholar, we screened the first 200 records ordered by relevance using broad combinations of dialysis, electrolyte and cardiovascular outcome terms.

Reference lists of all included articles and relevant reviews were manually searched to identify additional studies. Only full-text articles available in English and reporting adjusted hazard ratios (aHRs) for cardiovascular mortality or SCD in adult HD or PD populations were retained for quantitative synthesis. The complete database-specific search strategies, including all Boolean operators and field tags, are provided in Supplementary Table S1.

We also reviewed the reference lists of included studies and relevant review articles to identify any additional eligible studies not captured through the electronic database searches.

### 2.3. Inclusion and Exclusion Criteria

Studies were eligible if they enrolled adults (≥18 years) receiving maintenance HD, PD, or mixed-modality dialysis with extractable dialysis-specific data. Eligible exposures included serum electrolyte parameters: magnesium, chloride, sodium, calcium, phosphate, potassium, or related biochemical indices, reported either categorically or continuously. Studies were required to report cardiovascular mortality or SCD, including composite outcomes only when these endpoints were explicitly included, and to provide aHR with a 95% confidence interval using the most comprehensively adjusted model available. Only numerically reported effect estimates with clearly stated units, allowing protocol-specified unit conversion when needed, were accepted. All numerical values had to be directly extractable from tables, text, or labelled figures in the source documents, in accordance with the predefined extraction protocol.

Studies were excluded if they involved paediatric, transplant, conservatively managed chronic kidney disease (CKD) or acute kidney injury populations, or if dialysis-specific results were not separable in mixed cohorts. Studies reporting only all-cause, infectious, or non-cardiovascular outcomes, or composite outcomes lacking cardiovascular or SCD components, were excluded. Exposures unrelated to serum electrolytes (including dialysate-only concentrations without serum values) were not eligible. Studies without aHR and 95% confidence interval (Cl), reporting only unadjusted or non-HR effect measures, or providing interaction terms without standalone estimates were excluded. Reports with non-extractable numerical data (e.g., unlabelled figures, survival curves without HRs) or duplicate publications without unique data were also excluded. Finally, studies were excluded if exposure or reference groups were unclear or if HR/CI values failed protocol-mandated back-validation beyond the accepted threshold.

### 2.4. Data Extraction

**Figure 1:**
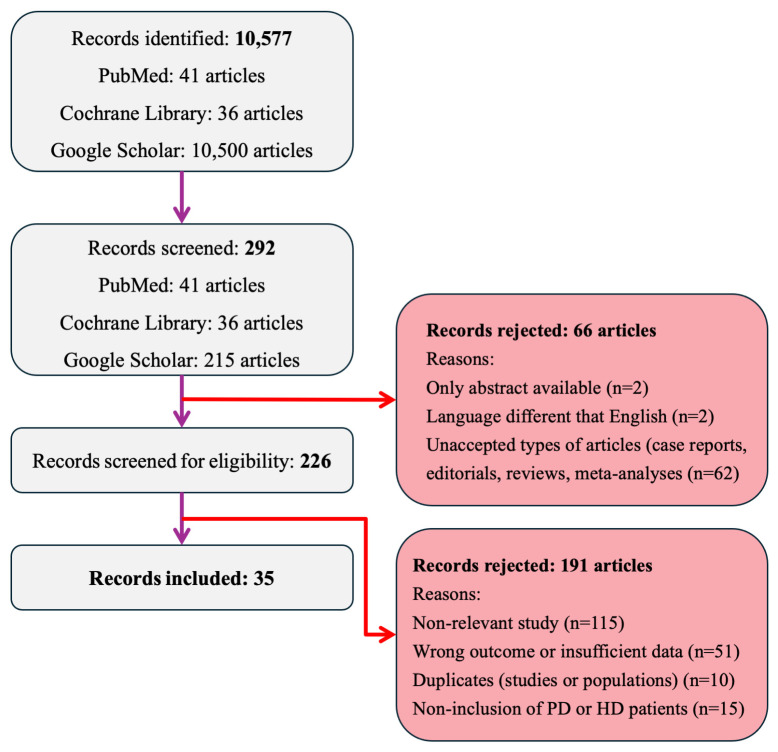
PRISMA Flow Diagram of Study Selection Process for Systematic Review.

All search results were imported into an Excel database and screened independently by two reviewers to remove duplicates and identify potentially relevant studies. Disagreements at either stage were resolved by discussion; if consensus was not reached, a third reviewer adjudicated (JR). Full texts were obtained for all records that passed initial screening; articles without accessible full texts were excluded. Each full-text report was then evaluated against the predefined eligibility criteria. Reasons for full-text exclusion were documented. The study selection procedure is summarized in the flow diagram (Figure 1). 

Data were extracted using a standardised, piloted extraction form. One reviewer extracted data and a second verified. author/year, country, study design, modality (HD/PD/mixed), sample size, population characteristics (age, sex, diabetes prevalence), exposure definition (baseline vs time-averaged; continuous vs categorical vs quantiles), comparator/reference category, outcome definition (cardiovascular mortality and/or SCD), follow-up duration, effect estimates (aHRs with 95% CIs), and covariates included in the most adjusted model. When studies reported multiple adjusted models for the same exposure–outcome pairing, we extracted the most fully adjusted model consistent with the prespecified primary exposure definition for that electrolyte.

### 2.5. Exposure Definitions

The exposures of interest were baseline serum concentrations of magnesium, potassium, calcium, phosphate, and sodium. Studies were included regardless of whether exposures were modelled as continuous variables, categorical thresholds, or quartiles, provided the comparisons were clinically interpretable and aligned with our pre-specified contrasts. When both continuous and categorical HRs were reported within a study, we selected one based on the most adjusted model and consistency with other studies in the same electrolyte–outcome pairing. Because different electrolytes have distinct physiological ranges, guideline targets, and expected risk patterns, we treated categorical, continuous, and quantile-based exposure models differently. For electrolytes with established clinical cut-offs and plausible non-linear (often U- or J-shaped) associations with cardiovascular events (magnesium, potassium, phosphate, calcium, chloride, and Ca × P), we treated categorical contrasts (e.g., low vs. normal vs. high) as the primary exposure definition whenever such data were available. When only continuous HRs were reported, these were used to characterise dose–response but were considered secondary to categorical contrasts.

For serum sodium, which in dialysis cohorts is typically reported over a relatively narrow range and was most often analysed as a continuous predictor, we treated per-unit continuous effects as the primary exposure definition, with categorical hyponatraemia vs. normonatraemia contrasts incorporated when directly comparable. For the Na/Cl ratio, where no universally accepted “normal” range exists and studies generally reported quantiles, we treated quartile-based contrasts as primary to allow non-linear risk patterns to be captured without imposing arbitrary thresholds.

When a single study reported more than one model (e.g., both categorical and continuous, or both baseline and time-varying exposures) for the same electrolyte–outcome pairing, we extracted all relevant estimates but included only one effect estimate per study in each pooled meta-analysis. Priority was given to the model that matched the primary exposure definition for that electrolyte (categorical, continuous, or quantile-based, as above) and used the most fully adjusted HR.

In all included cohorts, electrolyte measurements were obtained before the occurrence of cardiovascular events, most commonly as baseline values at cohort entry (e.g., at initiation of dialysis or at the first study visit) or as time-averaged values over an initial observation window, as defined by the original investigators. Individual patient-level intervals between a given electrolyte measurement and subsequent cardiovascular death or SCD were not reported in the source studies and could therefore not be reconstructed in this meta-analysis.

#### 2.5.1. Serum Magnesium

Serum magnesium was extracted as both categorical and continuous exposures, depending on how each study reported it. Categorical definitions most commonly contrasted hypomagnesaemia versus a normal range (e.g., <0.7 mmol/L vs. 0.7–1.2 mmol/L) or compared the lowest magnesium category with higher categories. These categorical contrasts formed the basis of the primary magnesium analyses in both PD and HD, because they align with clinical cut-offs and with prior evidence that risk is concentrated among patients with low magnesium. Continuous models (e.g., per 0.1 or 0.2 mmol/L higher magnesium) were extracted when available and were used as secondary or sensitivity analyses to explore the presence and approximate shape of any dose–response relationship.

#### 2.5.2. Serum Potassium

Potassium was primarily evaluated using predefined categorical ranges, typically with a mid-range category (approximately 4.0–4.5 mmol/L) as the reference and one or more categories reflecting hypokalaemia and hyperkalaemia (e.g., <3.5 mmol/L or ≥5.0 mmol/L). These categorical contrasts were chosen as the primary exposure definition because clinical practice and prior observational work in dialysis populations support a non-linear, U-shaped relationship between potassium and mortality or arrhythmic risk, with increased risk at both low and high levels. Continuous potassium models assuming a linear change in risk per mmol/L were less frequently reported and were not pooled when they conflicted with the clear categorical pattern or when data were too sparse; they were used descriptively when available.

#### 2.5.3. Serum Calcium

Serum calcium exposure was reported either as total calcium or albumin-corrected calcium (CaAlb) using study-specific normal ranges (for example, 2.10–2.37 mmol/L or 8.5–10.2 mg/dL). Comparisons included: low calcium: <2.10 mmol/L or <8.5 mg/dL, high calcium: >2.37 mmol/L or >10.2 mg/dL, or per-unit increases (e.g., per 1 mg/dL). When available, we extracted categorical contrasts corresponding to hypocalcaemia versus normocalcaemia and hypercalcaemia versus normocalcaemia. These categories were treated as primary because they reflect guideline-based targets and routine clinical decision thresholds. Some studies also provided continuous HRs (per mmol/L or per mg/dL higher calcium); these were extracted but, given the limited number of studies and heterogeneity in calcium measurement and correction, were not meta-analysed and were instead summarised narratively.

#### 2.5.4. Serum Phosphate

Phosphate was analysed using both categorical and continuous contrasts. Most studies defined categories relative to guideline-relevant ranges (for example, <1.13 mmol/L, 1.13–1.78 mmol/L, and >1.78 mmol/L, or analogous mg/dL cut-points), with the mid-range or recommended range as the reference category. These high-vs-reference categorical contrasts formed the basis of the primary phosphate analyses, in line with data that cardiovascular risk rises disproportionately at higher phosphate levels. When studies reported HRs per unit higher phosphate (e.g., per 1 mg/dL or per 0.3 mmol/L), we extracted these as continuous effects and treated them as secondary, using them to check whether the direction and approximate magnitude of the association were consistent with the categorical analyses. Quartile-based contrasts were incorporated when categorical cut-points did not align across studies but the relative ranking of exposure was still informative.

#### 2.5.5. Serum Sodium

Sodium was less frequently reported.

In HD cohorts, the most common approach was a continuous model, typically reporting HRs per 1 mEq/L higher baseline or time-averaged sodium. Because sodium values in dialysis patients generally lie within a narrow range and the available HD studies modelled sodium linearly, we treated these per-unit continuous effects as the primary sodium exposure definition in HD. Categorical definitions (e.g., hyponatraemia < 137 mEq/L vs. normonatraemia ≥ 139 mEq/L) were extracted where available and summarised, but were not always directly comparable across studies because of differing cut-points.

Several peritoneal dialysis cohorts reported the Na/Cl ratio rather than sodium alone, most often in quantiles (e.g., quartiles). For this exposure, we treated quartile-based contrasts, particularly highest vs. lowest quartile, as primary, because no universally accepted “normal” range for the Na/Cl ratio exists and the quantile approach allows potentially non-linear patterns of risk to be captured. When continuous HRs per unit higher Na/Cl ratio were reported, these were extracted as secondary estimates and used to check consistency with the quartile-based results.

#### 2.5.6. Chloride, Na/Cl Ratio and Ca × P Product

Serum chloride was reported in relatively few studies, usually using study-specific categorical thresholds (for example, <100, 100–103, >103 mmol/L). Because there are no established dialysis-specific chloride cut-offs for cardiovascular risk and continuous chloride models were rarely available, we used these categorical contrasts as the primary exposure definition for chloride, without attempting to impose a common threshold across studies.

The calcium–phosphate (Ca × P) product was extracted when reported as a separate exposure, typically as high vs. low or high vs. reference categories. Given the small number of contributing cohorts and differences in how Ca × P was calculated and categorised, we did not attempt to model Ca × P as a continuous variable. Instead, we used the published categorical contrasts to provide a qualitative and, where possible, quantitative summary of its association with cardiovascular outcomes.

#### 2.5.7. Outcome Definitions

The two primary outcomes were cardiovascular mortality—deaths from cardiac, vascular, or cerebrovascular causes—and SCD—defined as unexpected death due to cardiac causes within 1 h of symptom onset or within 24 h of being last seen alive and symptom-free.

When studies reported composite cardiovascular outcomes that included SCD, they were classified based on the outcome definition used by the original study.

For each electrolyte–outcome pairing, only one effect estimate per study was included to avoid duplication. When multiple models were available, we extracted the estimate from the most fully adjusted model to minimize confounding. If subgroup-only results were reported, we used the broadest and least restrictive subgroup consistent with the overall dialysis population.

### 2.6. Risk of Bias Assessment

Risk of bias of included cohort studies was assessed using the Newcastle–Ottawa Scale (NOS). Two reviewers evaluated each study independently, with disagreements resolved by discussion. The recorded NOS domain scores and derived an overall study judgement (e.g., low/moderate risk) are presented in Supplementary Table S2.

### 2.7. Certainty of the Evidence

We assessed certainty in the body of evidence for each main exposure-outcome association using [GRADE], considering risk of bias, inconsistency, indirectness, imprecision, and publication bias.

### 2.8. Statistical Analyses

All quantitative analyses were conducted using a predefined, fully reproducible workflow. Effect estimates were extracted as aHRs with 95% CIs from each eligible study. For all analyses, HRs were transformed to the logarithmic scale to ensure normality and permit pooling across heterogeneous studies. Standard errors (SEs) of the log-HRs were computed directly from the reported confidence intervals using the formula:

$${SE}_{ln(HR)}=\frac{\ln\left(upper\;CI\right)-\ln(lower\;CI)}{3.92}$$

For each electrolyte–outcome pairing we specified, in advance of pooling, which exposure model would be treated as primary. Categorical models were primary for magnesium, potassium, phosphate, calcium, chloride, and Ca × P (typically contrasting clinically abnormal categories with mid-range or guideline-recommended reference levels). Quartile-based models were primary for the Na/Cl ratio. Continuous models were primary for serum sodium in HD cohorts, where available studies modelled sodium per unit increase over a relatively narrow range. Continuous effects for other electrolytes (e.g., magnesium and phosphate) were extracted when at least two studies reported compatible scaling but were considered secondary to the categorical analyses. When a study contributed both categorical and continuous estimates for the same electrolyte–outcome combination, or both baseline and time-varying models, we included only one effect estimate in each meta-analysis, prioritising: the model that matched the primary exposure definition for that electrolyte, and the most fully adjusted model.

Log-transformed effect sizes and their SEs served as inputs for all meta-analytic models. The primary approach used random-effects meta-analysis with restricted maximum likelihood (REML) estimation to account for between-study heterogeneity. For each electrolyte and outcome (SCD or cardiovascular mortality), pooled HRs were back-transformed from log scale to produce interpretable summary estimates with 95% CIs.

Between-study variability was quantified using τ^2^ (tau-squared), I^2^ statistics, and Cochran’s Q test. When sufficient studies (≥10) were available, publication bias was assessed using funnel plots and Egger’s regression test. Predefined subgroup analyses were performed for haemodialysis (HD) and peritoneal dialysis (PD), and meta-regression was applied to evaluate modality-specific differences.

Sensitivity analyses included leave-one-out influence testing and comparison of fixed-effect versus random-effects models to evaluate the robustness of pooled estimates. All numerical calculations, data transformations, model fitting, subgroup analyses, and figure generation (forest plots, funnel plots) were executed using statistical program to ensure transparency and reproducibility.

#### Software and Figure Generation

All analyses were conducted using standard procedures implemented in the statistical software (IBM SPSS Statistics 29 software package, trial version). Figures were generated using built-in plotting and graphing functions within the same software, followed by minor formatting for presentation. No bespoke programming or standalone figure-generation scripts were developed beyond these standard commands. 

## 3. Results

### 3.1. Flowchart and Study Characteristics

The study selection process is given in Figure 1. In total, 10,577 records were identified through database searching (PubMed: *n* = 41; Cochrane CENTRAL: *n* = 36; Google Scholar: *n* = 10,500). After removal of duplicates, 292 unique records were screened by title and abstract. Of these, 66 were excluded at this stage (only abstract available, *n* = 2; language other than English, *n* = 2; non-eligible article types such as case reports, editorials, reviews, or meta-analyses, *n* = 62). The remaining 226 articles were assessed for eligibility based on full text. A further 191 studies were excluded because they were non-relevant to the review question (*n* = 115), reported wrong outcomes or insufficient data for extraction (*n* = 51), represented duplicate studies or populations (*n* = 10), or did not include patients on HD or PD (*n* = 15). Ultimately, 35 cohort studies met the inclusion criteria and were incorporated into the meta-analysis.

Collectively, these studies represented a large cohort of adult dialysis patients, with sample sizes ranging from small single-centre cohorts of fewer than 200 patients to large registry-based cohorts exceeding 140,000 participants. Most included cohorts were prospective or retrospective observational studies or analyses derived from national or multicentre dialysis registries.

Across the included studies, the average proportion of male participants was approximately 60%, although individual studies showed considerable variation. Ethnic composition was diverse but unevenly distributed: most studies originated from Asian populations, particularly Japanese and Chinese cohorts, whereas a smaller number of studies were conducted in Europe and North America or in mixed-ethnicity populations comprising White, Black, Asian and Hispanic patients. In terms of dialysis modality, the majority of studies were conducted exclusively among HD patients, a smaller subset enrolled only PD patients, and several cohorts included both HD and PD populations. This distribution reflects the predominance of HD-based research in the literature while still allowing modality-specific and combined analyses within the present meta-analysis.

Across these 35 cohorts, the mean age of participants was 61.2 years, with HD cohorts generally older (approximately 64 years) than PD cohorts (approximately 53 years). The average proportion of male participants was 58%, ranging from 36% to 78%. Diabetes was present in ~39% of participants overall, with a higher prevalence in HD cohorts compared with PD cohorts. Median follow-up duration across studies averaged about 52 months.

Baseline biochemical parameters were variably reported across cohorts. Mean serum sodium concentrations were typically within the physiologic range (≈137 mEq/L), whereas potassium levels averaged 4.5–4.7 mEq/L in HD and ~4.3 mEq/L in PD cohorts. Serum calcium values averaged 2.22 mmol/L, and phosphorus levels averaged 1.6 mmol/L, with PD cohorts showing slightly higher phosphate concentrations. Magnesium was reported in only a subset of studies, with an overall mean of approximately 1.24 mmol/L. Serum chloride data were sparse but generally fell near 101–102 mmol/L.

### 3.2. Electrolyte Associations in PD

In PD cohorts, several electrolyte abnormalities, particularly dyskalaemia, hypomagnesaemia and hyperphosphataemia, were associated with higher cardiovascular mortality, whereas data for calcium, sodium and chloride were limited to one or a few cohorts. We performed a modality-specific meta-analysis to assess the association between serum electrolyte abnormalities at baseline (or time-averaged, where reported) and the risk of cardiovascular mortality (with SCD included within cardiovascular death when so defined in the original study) in patients undergoing PD. A total of five PD cohorts contributed categorical comparisons reflecting severe potassium abnormalities (most often hypokalaemia, and in some instances marked hyperkalaemia) versus mid-range reference values [31,32,33,34,35]. The pooled random-effects estimate demonstrated a significant association between potassium abnormalities and higher cardiovascular mortality. The pooled HR for the most extreme potassium category versus reference was 2.05 (95% CI 1.32–3.20; *p* = 0.002). Heterogeneity was high (I^2^ > 80%), reflecting divergent potassium cut-offs, differences in measurement frequency, and regional variation (Asian vs. non-Asian cohorts). Leave-one-out analyses showed consistently elevated risk across all combinations (pooled HR range 1.56–2.26), indicating that the direction of effect was robust despite variability in magnitude between cohorts. In exploratory subgroup analyses by geographic region, both Asian (China; *n* = 2) and non-Asian (Brazil and USA; *n* = 3) cohorts demonstrated increased risk. The pooled HR in Asian studies was 1.85 (95% CI 1.14–2.99; I^2^ = 0%), whereas non-Asian studies showed a similar point estimate but significantly greater variability (HR = 2.16; 95% CI 1.15–4.04; I^2^ = 91%), indicating that heterogeneity was concentrated in non-Asian settings.

Two studies were included in the primary magnesium analysis, each comparing low versus normal serum magnesium [36,37]. The pooled categorical HR was 1.73 (95% CI 1.23–2.44; *p* = 0.002), indicating a statistically significant increase in cardiovascular mortality among patients with hypomagnesaemia. Heterogeneity was minimal (I^2^ = 0%, τ^2^ = 0.000, Q = 0.57, df = 1, *p* = 0.45), suggesting that the effect of low magnesium was highly consistent across PD cohorts. In contrast, the two studies reporting continuous magnesium effects showed very high between-study heterogeneity (HR = 0.14; 95% CI 0.00–5.36; *p* = 0.29; τ^2^ = 6.50; Q = 17.59, df = 1; *p* < 0.001; I^2^ = 94%) [36,38]. Differences in exposure scaling (mg/dL vs. mmol/L) and exposure modelling combined with the limited number of effect estimates rendered these results unreliable for clinical interpretation. Therefore, emphasis was placed on the consistent categorical finding that hypomagnesaemia is associated with substantially increased cardiovascular mortality risk.

Four studies contributed categorical phosphate contrasts, typically comparing high phosphate ranges with mid-range reference categories [39,40,41,42]. Elevated phosphate was significantly associated with increased cardiovascular mortality (HR = 2.03; 95% CI 1.20–3.43; *p* = 0.008). Categorical phosphate also showed high heterogeneity (I^2^ ≈ 79%), although all studies demonstrated increased risk with higher phosphate concentrations. Continuous phosphate models (two cohorts) did not support a linear dose–response relationship (HR = 1.01; 95% CI 0.62–1.62; *p* = 0.98) [41,42]. Taken together, these findings suggest that cardiovascular risk may be disproportionately concentrated at the highest phosphate levels rather than increasing uniformly with incremental changes.

Data for calcium alone were insufficient to permit a pooled analysis. However, one PD cohort provided categorical results for the calcium × phosphate product (Ca × P) [42]. In that study, higher Ca × P was strongly associated with cardiovascular mortality (high vs. low Ca × P category HR = 2.17; 95% CI 1.45–3.26; *p* < 0.001), suggesting that the combined mineral load of calcium and phosphate may be an important determinant of cardiovascular risk in PD, even though the independent effect of calcium could not be quantified in our meta-analysis [43].

The chloride, sodium, and Na/Cl ratio analyses were each based on a single cohort (k = 1) [44,45,46]; therefore, heterogeneity could not be assessed (I^2^ = 0; τ^2^ = 0 by definition) and findings were interpreted as study-specific observations rather than pooled estimates. Lower serum chloride was associated with higher cardiovascular mortality: chloride <100 mmol/L had an approximately 2.86-fold higher risk of cardiovascular death compared with >103 mmol/L, and even moderately reduced chloride (100–103 mmol/L) remained at significantly elevated risk [44]. Time-varying analyses showed similar patterns, with both chloride 97–101 mmol/L and <97 mmol/L associated with ~1.44–1.49-fold higher cardiovascular mortality vs. >101 mmol/L, suggesting that persistent hypochloraemia is detrimental. By contrast, a higher Na/Cl ratio was strongly and independently associated with lower cardiovascular mortality. Compared with the lowest Na/Cl quartile (<1.33), patients in the highest quartile (>1.42) had a 62% lower risk of cardiovascular death (HR 0.38, 95% CI 0.22–0.67), and the continuous model showed a 40% risk reduction per 0.1 unit increase in Na/Cl (HR 0.60, 95% CI 0.46–0.78) [45]. These findings suggest that chloride-related indices, particularly the Na/Cl ratio, may capture acid–base and volume-related risk in PD more sensitively than sodium alone. However, as each marker was evaluated in a single cohort, these results should be interpreted as hypothesis-generating and require replication. In PD, evidence for sodium as an independent cardiovascular risk factor was limited. In a single PD cohort, the categorical comparison of 137–<139 mEq/L vs. ≥139 mEq/L yielded a wide CI (HR 1.37, 95% CI 0.64–2.92), indicating substantial imprecision and preventing any definitive conclusion regarding the association between serum sodium and cardiovascular mortality [46]. Similarly, the continuous sodium model (per 1 mEq/L higher) produced an imprecise estimate (HR 0.95, 95% CI 0.84–1.08) that did not reach statistical significance.

**Figure 2:**
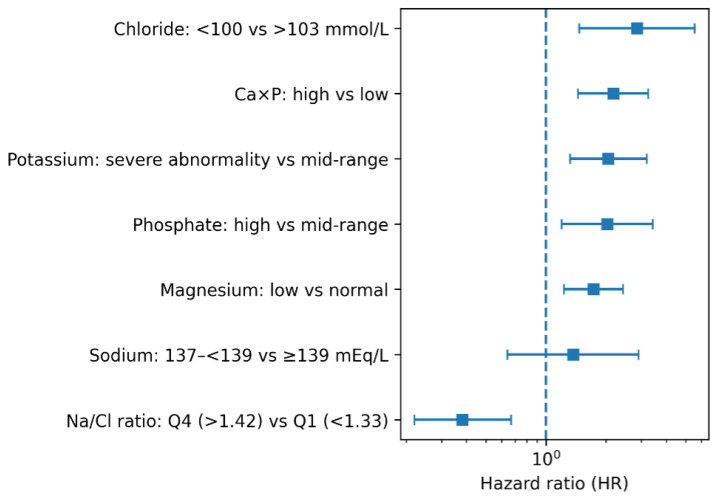
Summary forest plot of serum electrolyte abnormalities and cardiovascular mortality in peritoneal dialysis.

All modality-specific associations are summarised in Table 1 and Figure 2.

### 3.3. Electrolyte Associations in Haemodialysis (HD)

In HD cohorts, hypomagnesaemia and hyperphosphataemia emerged as the most consistent adverse markers of cardiovascular mortality, with lower serum sodium also associated with increased risk, whereas evidence for potassium and other electrolytes was more limited. We evaluated the association between baseline or time-averaged serum electrolyte profiles and cardiovascular mortality (with SCD included when defined as part of cardiovascular death in the original study). After excluding non-cardiovascular outcomes, explicit subgroup strata, and variability-based measures, the main HD analysis consisted of studies reporting aHR for categorical or continuous electrolyte exposures.

Three HD cohorts reported categorical comparisons of low versus normal serum magnesium [47,48,49]. The pooled random-effects estimate showed a significantly higher risk of cardiovascular mortality among patients with hypomagnesaemia (HR = 2.17, 95% CI 1.17–4.00; *p* = 0.011). Between-study heterogeneity was low (I^2^ = 26%, τ^2^ = 0.085; Q = 2.72, df = 2, *p* = 0.26), suggesting a broadly consistent association across settings. Leave-one-out analyses yielded pooled HRs ranging from 1.94 to 2.46, with all 95% CIs excluding unity, indicating that the direction and magnitude of the association were not driven by any single study. Continuous magnesium models were highly heterogeneous and produced unstable pooled estimates, precluding reliable interpretation; therefore, clinical inference was based on the categorical hypomagnesaemia contrasts.

Three HD studies contributed categorical comparisons of high versus reference phosphate categories [39,47,50]. Elevated phosphate was associated with a marked increase in cardiovascular mortality (HR = 1.98, 95% CI 1.36–2.68; *p* < 0.001). Heterogeneity was substantial (I^2^ = 79.5%, τ^2^ = 0.051; Q = 9.77, df = 2, *p* = 0.008), reflecting variation in the definition of “high” phosphate (different mg/dL or mmol/L thresholds), the use of baseline versus time-averaged exposure, and differences in covariate adjustment. Leave-one-out analyses did not materially alter the pooled effect (HR range 1.72–2.23), although heterogeneity persisted, suggesting that the direction of association is robust, but its magnitude may be population-specific.

Two HD cohorts provided categorical contrasts comparing extreme potassium levels (usually hypokalaemia or severe hyperkalaemia) with mid-range reference categories [51,52]. The pooled random-effects estimate did not demonstrate a statistically significant association with cardiovascular mortality (HR = 1.13, 95% CI 0.68–1.88; *p* = 0.63). Heterogeneity was modest (I^2^ = 29.8%, τ^2^ = 0.065; Q = 1.42, df = 1, *p* = 0.23) consistent with a weak and statistically non-significant pooled effect. With only two contributing studies, sensitivity analyses and subgroup exploration were limited, and the null result should be interpreted cautiously.

Three HD studies reported continuous models for serum sodium [18,25,53]. When pooled, higher sodium concentrations were consistently associated with lower cardiovascular mortality (HR per unit increase = 0.94, 95% CI 0.91–0.96; *p* < 0.001). There was no evidence of heterogeneity (I^2^ = 0%, τ^2^ = 0.000; Q = 0.76, df = 2, *p* = 0.68), suggesting that the inverse association between sodium and cardiovascular death was highly consistent across HD cohorts despite differences in patient characteristics and adjustment sets. This pattern suggests that lower sodium, potentially reflecting hyponatraemia, volume overload, or poor nutritional status, is associated with increased cardiovascular risk in HD.

Overall, in the HD population, categorical hypomagnesaemia and elevated phosphate levels were robustly associated with excess cardiovascular mortality, whereas potassium abnormalities did not show a clear pooled effect and continuous sodium levels exhibited a modest but consistent inverse association with risk.

**Figure 3:**
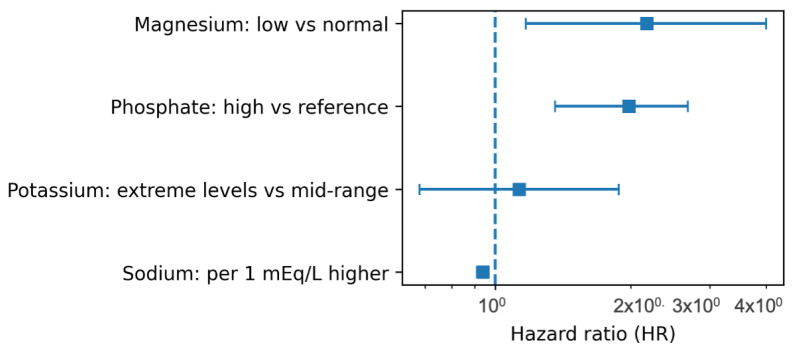
Summary forest plot of serum electrolyte abnormalities and cardiovascular mortality in haemodialysis.

All modality-specific associations are summarised in Table 2 and Figure 3.

### 3.4. Electrolyte Associations in All Dialysis Patients Combined (PD + HD)

When data from PD and HD cohorts were pooled, hyperphosphataemia, hypomagnesaemia and extreme potassium abnormalities showed the clearest associations with cardiovascular mortality, while higher sodium concentrations were modestly protective in continuous models. Across all included HD and PD cohorts, abnormalities in several serum electrolytes were significantly associated with cardiovascular mortality when studies were pooled irrespective of dialysis modality. For most electrolytes, categorical exposure contrasts consistently demonstrated the strongest associations.

Across six studies (seven categorical contrasts), high phosphate levels were associated with a significantly elevated risk of cardiovascular mortality (pooled HR = 2.09, 95% CI 1.60–2.72; *p* < 0.001) [39,40,42,47,50,54]. Between-study heterogeneity was substantial (I^2^ ≈ 79%, τ^2^ = 0.085), reflecting differences in phosphate thresholds, time-averaged versus baseline exposures, and adjustment strategies. Despite this, the direction of association was uniform, with all studies indicating increased cardiovascular risk at higher phosphate concentrations.

Five studies evaluating categorical magnesium levels showed that low magnesium was consistently associated with higher cardiovascular mortality (pooled HR = 1.82, 95% CI 1.39–2.38; *p* < 0.001), with no detectable heterogeneity (I^2^ = 0%) indicating a highly consistent elevation in cardiovascular risk with abnormal magnesium [36,37,48,49,55]. Conversely, continuous models demonstrated an inverse relationship (HR = 0.68 per unit increase; 95% CI 0.50–0.93; *p* = 0.015), but with marked heterogeneity (I^2^ ≈ 82%) due to incompatible units and exposure scaling [13,36,38,55,56,57]. Thus, categorical contrasts provide the most clinically interpretable evidence of increased cardiovascular risk with hypomagnesaemia.

Seven categorical potassium contrasts showed that extreme potassium abnormalities were associated with increased cardiovascular mortality (pooled HR = 1.73, 95% CI 1.22–2.46; *p* = 0.002) [32,33,34,35,51,52,58]. Heterogeneity was high (I^2^ ≈ 81%) and largely attributable to known modality-specific biological differences: PD cohorts consistently demonstrated strong positive associations, whereas HD studies showed weaker or null effects. In continuous models (four studies), each unit increase in serum sodium was associated with a significant reduction in cardiovascular mortality (HR = 0.94, 95% CI 0.92–0.96; *p* < 0.001), again with I^2^ = 0%, with nearly identical leave-one-out estimates, reinforcing its stability as an inverse predictor of cardiovascular mortality in dialysis patients [18,25,46,53].

Overall, these combined results demonstrate that electrolyte abnormalities, particularly high phosphate, low magnesium, abnormal potassium, and dysnatremia, are key, consistent markers of cardiovascular mortality across dialysis modalities.

**Figure 4:**
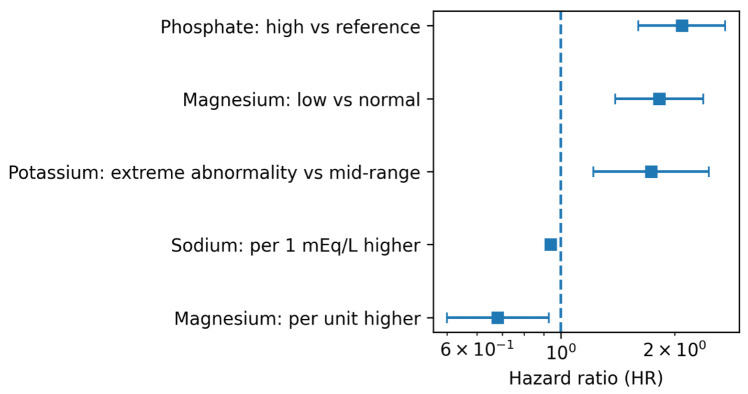
Combined analysis (HD + PD): summary forest plot of serum electrolyte abnormalities and cardiovascular mortality.

All modality-specific associations are summarised in Table 3 and Figure 4.

### 3.5. Heterogeneity, Sensitivity Analyses, and Risk of Bias Across HD, PD and Combined Modes

Across all electrolytes and both dialysis modalities, the degree of between-study heterogeneity varied substantially and was strongly dependent on the electrolyte assessed, the exposure definition (categorical vs. continuous), and whether studies were conducted in HD, PD or combined analyses. High phosphate and abnormal magnesium consistently demonstrated statistically significant associations with cardiovascular mortality across modalities; however, the magnitude and pattern of heterogeneity differed between HD, PD, and pooled analyses.

#### 3.5.1. Sensitivity Analyses Across Modalities

Sensitivity analyses were performed for all electrolyte–modality combinations with at least three available effect estimates. Across HD, PD and combined analyses, the direction of association for key electrolytes (phosphate, magnesium, potassium) was highly stable. For categorical magnesium, exclusion of any single study did not materially alter pooled estimates, and all leave-one-out hazard ratios remained >1.0. Similarly, for categorical phosphate, every leave-one-out model preserved the direction of effect, and all confidence intervals excluded unity, despite substantial heterogeneity. For PD potassium, leave-one-out analyses consistently showed elevated cardiovascular risk, though absolute effect sizes varied widely, indicating robust directionality but marked scale heterogeneity. Continuous sodium in HD showed virtually identical leave-one-out estimates, with persistent I^2^ = 0%, confirming exceptional reproducibility. By contrast, sensitivity analyses for continuous magnesium and continuous phosphate failed to resolve the extreme heterogeneity observed in primary models, underlining the greater interpretability and clinical coherence of categorical contrasts. Additional sensitivity checks, excluding studies with high risk of bias, unclear comparator groups, or possible cohort overlap, did not materially change pooled HRs or heterogeneity metrics, further affirming the robustness of the primary findings. For electrolytes with limited study numbers (e.g., calcium-related markers, sodium categorical analyses), sensitivity analyses had limited power, but no single study had a disproportionate influence on results.

#### 3.5.2. Risk of Bias and Publication Bias

Methodological quality was assessed using the Newcastle–Ottawa Scale (NOS). Overall, all studies were classified as moderate quality according to predefined NOS thresholds. No study was excluded on the basis of methodological quality (Supplementary Table S2).

The number of studies per electrolyte (typically 2–7) prevented reliable formal evaluation of publication bias, as funnel plots and regression-based asymmetry tests are not recommended when <10 effect sizes are available. As such, Egger’s and Begg’s tests were not conducted. Nevertheless, the consistent directional stability observed across all sensitivity analyses, particularly for categorical phosphate, magnesium, and PD potassium, reduces the likelihood that the observed associations were driven solely by small-study effects or selective reporting. For electrolytes with weak or inconsistent pooled effects (HD potassium, continuous magnesium, continuous phosphate), publication bias cannot be excluded due to limited data and greater statistical instability.

Overall, moderate to substantial heterogeneity was observed for several electrolytes, but sensitivity testing indicated that this represented genuine clinical or methodological variability rather than instability of the underlying associations. The combination of stable effect direction, robustness to exclusion of individual studies, and consistency across modalities supports the reliability of the core findings.

Certainty was low/very low for included studies, mainly due to limited number of studies and imprecision.

## 4. Discussion

Cardiovascular death, and particularly SCD, is the dominant cause of mortality in dialysis, accounting for roughly one quarter of deaths in HD cohorts and a substantial proportion of deaths across end-stage kidney disease populations [2,59]. Our meta-analysis expands existing evidence that specific serum electrolyte abnormalities are associated with cardiovascular mortality, including SCD, in dialysis patients, with visible modality-specific differences, particularly for potassium. Because all included studies were observational cohorts, the electrolyte–outcome associations we identified should primarily be interpreted as risk markers rather than proven causal mediators. The observed associations are compatible with a potential causal contribution of specific electrolyte disturbances to arrhythmogenesis and cardiovascular instability, but causality cannot be established from these analyses. Residual confounding by nutritional status, systemic inflammation, comorbidity burden, dialysate composition and prescription patterns (including dialysate baths, ultrafiltration and concomitant medications) may partly explain both the magnitude and the modality-specific differences in associations.

### 4.1. Practical Clinical Implications by Dialysis Modality

#### 4.1.1. Peritoneal Dialysis

In PD, categorical analyses showed that severe potassium abnormalities, low serum magnesium, high serum phosphate and a low Na/Cl ratio were all associated with substantially higher cardiovascular mortality, whereas data for calcium alone were sparse. Typically, the highest-risk categories for potassium, magnesium and phosphate were associated with about a two-fold increase in cardiovascular mortality compared with mid-range reference categories, and a lower Na/Cl ratio identified patients at particularly high risk. Clinically, these findings support a PD strategy that systematically screens for and corrects chronic hypokalaemia (through diet, dialysate composition and review of medications), maintains phosphate within guideline targets using diet, phosphate binders and optimisation of dialysis adequacy, and avoids unnecessary lowering of magnesium in patients at increased arrhythmic risk [40,60].

Low chloride and a low Na/Cl ratio should be interpreted as additional markers of unfavourable acid–base and volume status and of possible malnutrition–inflammation, prompting a broader review of fluid management, peritonitis risk, nutritional support and cardiometabolic therapy, rather than treatment of the electrolyte abnormality in isolation [25,61].

#### 4.1.2. Haemodialysis

In HD cohorts, categorical hypomagnesaemia and hyperphosphataemia emerged as the most consistent adverse markers of cardiovascular mortality, and lower serum sodium in continuous models showed a modest but monotonic association with higher risk, whereas categorical potassium abnormalities did not show a statistically robust pooled effect. In practice, these data support an HD approach that prioritises tight control of phosphate (diet, binders and dialysate prescription) to limit calcific and structural cardiovascular damage, recognises low magnesium as a high-risk marker and is cautious about interventions that further reduce magnesium in patients with arrhythmic vulnerability, and identifies and treats hyponatraemia or low sodium early, understanding that this often reflects volume overload, heart failure or malnutrition–inflammation.

Potassium management in HD should continue to focus on avoiding both chronic extremes and large serum-dialysate gradients via individualised potassium baths and careful ultrafiltration, even though the pooled categorical associations with cardiovascular mortality were less clear than in PD [23,62,63]. Overall, routine electrolyte profiles in both modalities should be viewed as integrated markers of cardiovascular vulnerability that can help refine risk stratification and guide a more holistic review of dialysis prescription and supportive care [52,63].

### 4.2. Potassium: Modality-Specific Patterns and Arrhythmic Risk

Our finding that extreme potassium categories in PD were associated with a twofold higher risk of cardiovascular mortality, with directionally stable leave-one-out analyses, is concordant with PD literature implicating hypokalaemia as a major driver of cardiac events in this modality. In the PDOPPS international cohort, hypokalaemia was common and independently associated with higher all-cause mortality and peritonitis, with a marked proportion of excess deaths being cardiovascular [64]. Large PD datasets such as the DaVita cohort have reported a U-shaped relationship between time-averaged serum potassium and both all-cause and CV mortality, with increased risk at levels < 3.5 mEq/L and ≥5.5 mEq/L [33]. A systematic review showed that hypokalaemia was significantly associated with both all-cause and cardiovascular mortality in PD cohorts [65]. Earlier single-centre reports suggested that hypokalaemia may “disproportionately” account for the high cardiac risk in PD through fatal ventricular arrhythmias [66,67,68].

At the cellular level, changes in extracellular potassium directly modulate cardiac potassium currents and the Nernst potential for potassium, altering resting membrane potential and action potential morphology. The inward-rectifier potassium current (I_K1_), carried mainly by Kir2.x channels, is crucial for stabilising the diastolic membrane potential and shaping terminal repolarisation of ventricular myocytes [69,70]. Hypokalaemia reduces the conductance of I_K1_ and several delayed-rectifier currents (especially I_Kr_), and inhibits Na^+^, K^+^ ATPase, thereby reducing repolarisation reserve, prolonging action potential duration and QT interval, and favouring early after-depolarisations and torsades-de-pointes–type ventricular tachyarrhythmias [71,72,73]. Conversely, hyperkalaemia depolarises the resting potential, partially inactivates fast sodium channels, slows conduction velocity and promotes conduction block or asystole, particularly in the presence of structural heart disease [71,73]. Both conduction block and asystole are recognised triggers of SCD in ESKD [62,74]. Genovese et al. [75] highlighted the arrhythmogenic potential of both hypo- and hyperkalaemia and the important role of potassium fluctuations in SCD among dialysis patients. In turn, Schuettler et al. [76] reported that potassium fluctuations during HD were associated with periodic repolarization dynamics (PRD), suggesting that substantial potassium removal may heighten the influence of efferent sympathetic activity on ventricular repolarisation and thereby contribute to malignant arrhythmias and SCD. Taken together, these data and our pooled estimates indicate that chronic low potassium in PD is a marker of cardiovascular vulnerability and may contribute to SCD risk, although we cannot exclude substantial confounding by malnutrition, inflammation and prescription factors.

In contrast, the pooled HD analysis did not show a statistically significant association between extreme potassium categories and cardiovascular mortality, despite a trend toward harm. This is compatible with large HD cohorts which report a U-shaped association of potassium with all-cause mortality, but less consistent and less specific effects on cardiovascular death once confounding by comorbidity, dialysate potassium, and potassium gradients is taken into account [62]. Case–control studies focusing specifically on SCD in HD have implicated both high pre-dialysis potassium and large serum-dialysate potassium gradients as triggers of cardiac arrest, but these relationships are highly time-dependent and may not be captured by static baseline measures [3,9]. Our results therefore suggests that chronic hypokalaemia in PD, where dialysate potassium is typically zero and clearance is continuous, is more tightly linked to structural and electrophysiological myocardial vulnerability than the intermittent potassium shifts that characterise HD. In HD, arrhythmic risk appears to depend at least as much on the dialysis prescription (potassium bath, ultrafiltration, session length) as on baseline serum levels [2].

### 4.3. Magnesium as a Consistent Prognostic Marker

The consistent association between hypomagnesaemia and cardiovascular mortality in both PD and HD in our categorical analyses aligns closely with growing evidence that magnesium is a key modifier of arrhythmic risk and vascular health in kidney failure. Magnesium exerts predominantly anti-arrhythmic effects by modulating calcium and potassium currents. Experimental and clinical data indicate that Mg^2+^ suppresses early after-depolarisations by inhibiting L-type Ca^2+^ current and modulating delayed-rectifier K^+^ currents, thereby decreasing dispersion of ventricular repolarisation [77,78]. Hypomagnesaemia therefore amplifies triggered activity and predisposes to torsades de pointes and other polymorphic ventricular tachyarrhythmias, consistent with the established efficacy of intravenous magnesium in terminating such arrhythmias [78,79]. The roughly twofold higher cardiovascular mortality we observed with low magnesium in both PD and HD is thus physiologically plausible and likely reflects a combination of impaired repolarisation stability, increased susceptibility to early after-depolarisations and broader vascular effects. In a Chinese PD cohort, Cai et al. [56] observed that patients with serum magnesium < 0.7 mmol/L had significantly higher all-cause and cardiovascular mortality over a median follow-up of 29 months, with magnesium emerging as an independent negative predictor of cardiovascular death after multivariable adjustment. A second PD cohort with a 5-year follow-up similarly found that lower magnesium concentrations were associated with higher cardiovascular mortality and greater vascular calcification [36]. Comparable findings have been reported in HD, where multiple large cohorts from the United States, Europe and Asia have shown that low or even low-normal magnesium is associated with higher all-cause and cardiovascular mortality, independent of traditional risk factors, with some suggesting a J-shaped relationship at very high magnesium levels [15,80,81]. In a European HD cohort, van Zuijdewijn et al. [13] demonstrated that higher magnesium levels were associated with significantly lower risk of sudden death, supporting a direct anti-arrhythmic effect. Taken together, these results support hypomagnesaemia as a strong prognostic marker of cardiovascular risk, consistent with a potential causal contribution, but they cannot distinguish whether low magnesium is a direct mediator of arrhythmic and vascular risk or a surrogate for broader processes such as protein–energy wasting, systemic inflammation, comorbidity burden or dialysate magnesium prescription.

### 4.4. Phosphate and Ca × P: Mineral Load and Cardiovascular Mortality

Hyperphosphataemia emerged from our analysis as one of the strongest and most consistent electrolyte predictors of cardiovascular mortality, with approximately a twofold increase in risk at the highest phosphate categories in both PD and HD and a similar pooled effect when modalities were combined. This aligns with extensive evidence from CKD and dialysis cohorts linking higher phosphate levels to vascular calcification, left ventricular hypertrophy, and increased cardiovascular events [82,83,84]. In PD, Huo et al. [85] showed that longer time in target phosphorus range during the first year of PD was associated with substantially lower risks of both all-cause and cardiovascular mortality. In turn, Huang et al. reported that combinations of low albumin with either high or low phosphorus identified PD patients at particularly high risk of cardiovascular mortality [41]. Serial analyses in PD have further confirmed that persistent phosphorus levels above guideline targets predict morbidity and death [86,87]. Our PD-specific pooled hazard ratio for the highest phosphate categories agrees with these observations and, by focusing on cardiovascular mortality, emphasises that the principal excess risk at high phosphate ranges is cardiovascular rather than infectious or malignancy-related. In HD, the association between hyperphosphataemia and cardiovascular mortality extends beyond atherosclerotic events to SCD specifically. The Q-Cohort, a large Japanese HD registry with ten years of follow-up, showed that higher serum phosphate was independently associated with an elevated risk of sudden death after adjustment for a wide range of confounders [88,89]. Other analyses and reviews have reinforced hyperphosphataemia as a central driver of arrhythmogenic substrate, mediated through extensive vascular and valvular calcification, increased arterial stiffness, which increase dispersion of repolarisation and create a substrate for re-entrant ventricular tachyarrhythmias [82,90,91,92,93]. Our hazard ratios are therefore consistent with these long-term datasets and suggest that hyperphosphataemia may be particularly important in determining which patients succumb to SCD rather than non-sudden cardiovascular death. The lack of a linear signal in continuous models supports the notion that risk is disproportionately concentrated in the upper tail of the phosphate distribution. Nonetheless, because phosphate levels are closely related to dietary intake, nutritional status, inflammation, dialysis adequacy, dialysate calcium/phosphate prescriptions and use of phosphate binders, our meta-analysis cannot determine whether phosphate acts primarily as a causal mediator or as an integrated marker of these interrelated factors.

The single PD cohort in our dataset that reported on calcium–phosphate product found that higher Ca × P was strongly associated with cardiovascular mortality, with hazard ratios comparable in magnitude to those seen for phosphate alone. This aligns with results of dialysis cohorts in which elevated Ca × P product has been associated with increased risks of cardiovascular death and SCD [82,90,94]. These findings, together with well-described mineral-driven vascular and valvular changes, are consistent with the strong associations we observed between hyperphosphataemia, elevated Ca × P and cardiovascular mortality, and support the view that a substantial part of this signal likely reflects fatal arrhythmias. Although our meta-analysis could not separately quantify the independent effect of calcium, these findings support KDIGO recommendations that consider Ca × P rather than phosphate in isolation, when managing mineral bone disorder in dialysis, and suggest that extreme Ca × P values may identify patients at particularly high risk of fatal arrhythmias. Large randomized trials comparing calcium-based with non-calcium-based phosphate binders have not shown clear mortality benefit [95]. Elevated Ca × P should therefore be regarded as a high-risk marker of cumulative mineral load rather than proof that lowering Ca × P per se will reduce cardiovascular events; interventional data are still required to establish causality.

### 4.5. Sodium, Chloride and Na/Cl Ratio

The results of our sodium analyses highlight another axis where modality and exposure definition matter. In HD, we observed a modest but consistent inverse association between serum sodium and cardiovascular mortality in continuous models, with each 1 mEq/L higher sodium associated with roughly 6% lower risk and no detectable heterogeneity between cohorts. This pattern is in line with several large HD studies showing that low pre-dialysis sodium and hyponatraemia are independently associated with higher all-cause and cardiovascular mortality and major cardiovascular events [18,19,96,97]. Sun et al. found that each 4 mmol/L increase in baseline sodium was associated with a 19–28% reduction in all-cause death in maintenance HD [18,96]. Mechanistically, low sodium in HD may reflect volume overload, heart failure, or malnutrition–inflammation, all of which predispose to SCD through myocardial fibrosis, chamber dilation, and autonomic imbalance [98,99]. Alterations in extracellular sodium primarily act through changes in the Na^+^ gradient and effects on conduction and Na^+^/Ca^2+^ exchanger. Hyponatraemia can promote intracellular Ca^2+^ overload via reverse-mode NCX activation, which facilitates delayed after-depolarisations and arrhythmias [92,100,101].

In PD, our modality-specific sodium analysis was underpowered, but existing PD cohorts indicate that hyponatraemia is clinically important: lower serum sodium and time-averaged hyponatraemia have been associated with higher all-cause mortality and new-onset cardiovascular events, and PD patients with sodium < 140 mEq/L have higher mortality than those with 140–<142 mEq/L [46,102,103]. The absence of a statistically significant PD signal in our sodium analysis likely reflects study design rather than biological neutrality. However, because hyponatraemia and low sodium in dialysis are tightly coupled to volume overload, heart failure, malnutrition–inflammation and prescription factors such as dialysate sodium concentration and ultrafiltration targets, our findings indicate that low sodium is a powerful prognostic marker of cardiovascular and SCD risk, but cannot establish that sodium itself is the causal mediator of these outcomes.

The chloride and Na/Cl ratio findings in PD are particularly noteworthy because relatively few studies have addressed these markers directly. Our meta-analysis, based on a single large cohort, found that lower chloride categories and lower Na/Cl ratios were associated with higher cardiovascular mortality, with the Na/Cl ratio showing a strong inverse association with risk. Zhou et al. [25] reported higher cardiovascular and all-cause mortality in PD patients in the highest chloride quartile, with risk increasing across quartiles. In contrast, You et al. [45] showed that in PD found that higher baseline serum Na/Cl ratio was associated with lower all-cause and cardiovascular mortality, suggesting that the balance between sodium and chloride rather may better capture relevant acid–base and volume status that influences cardiovascular risk. Outside PD, several studies in heart failure, hypertension, and HD have linked low chloride to adverse outcomes, suggesting potential U-shaped or context-dependent effects of chloride in advanced cardiovascular and renal disease [24,104]. Our pooled results, which identify the Na/Cl ratio as a more robust inverse marker of cardiovascular mortality than sodium alone, support the concept that chloride-related indices may integrate information on volume expansion, metabolic acidosis, and neurohormonal activation that is directly relevant to SCD risk in PD. Given the single-cohort basis for these analyses and the close relationship between chloride indices, nutritional status, acid–base balance, dialysate composition and volume management, these chloride-related measures should currently be interpreted as hypothesis-generating risk markers, not as proven causal mediators.

### 4.6. Integrated Interpretation Across Modalities

When PD and HD data were pooled irrespective of modality, our categorical analyses demonstrated that high phosphate, low magnesium, and extreme potassium abnormalities were each associated with roughly 1.7- to 2.1-fold increases in cardiovascular mortality, while categorical sodium abnormalities and lower continuous sodium levels were associated with significantly higher cardiovascular risk. These combined results resonate with meta-analyses in broader CKD and dialysis populations showing that hyperphosphataemia, hypomagnesaemia, hypokalaemia and hyponatraemia are each independently associated with both all-cause and cardiovascular mortality [81,83,84]. Importantly for SCD, many of the included studies either reported sudden death explicitly (as in the Q-Cohort phosphate analysis and the European HD magnesium study) or contributed to composite cardiovascular endpoints in which SCD is known to account for a substantial proportion of events [2,13,88]. It is therefore plausible that a considerable share of the cardiovascular mortality signal in our meta-analysis reflects fatal arrhythmias rather than only progressive heart failure or atherothrombotic events.

Available studies reinforce the concept, already present in reviews of SCD in dialysis that electrolyte disturbances are not merely laboratory abnormalities but central components of the arrhythmogenic milieu in kidney failure [2]. Our work adds to this by showing that, in both PD and HD, static or time-averaged measures of phosphate, magnesium, potassium and sodium carry substantial prognostic information for cardiovascular death, and likely SCD, across diverse populations and healthcare settings. They also highlight that modality matters. Hypokalaemia appears particularly dangerous in PD, hyperphosphataemia is a potent predictor of SCD in HD and hypomagnesaemia is a consistently adverse marker in both modalities. Moreover, dysnatremia, including low Na/Cl ratio, signals heightened cardiovascular vulnerability, especially in HD. From a clinical perspective, this supports an approach to SCD prevention in dialysis that goes beyond device therapy and conventional cardiology risk factors to include meticulous management of dialysate composition, dietary intake, binder and magnesium supplementation strategies, and dynamic volume control aimed at maintaining key electrolytes within modality-appropriate target ranges. At the same time, the heterogeneity we observed, particularly for potassium and chloride, points to the need for prospective, interventional studies that test whether correcting specific electrolyte perturbations can actually reduce cardiovascular and sudden death events, an evidence gap that remains largely unfilled despite decades of observational work.

## 5. Strengths and Limitations

This study has several notable strengths. It represents the first meta-analysis to systematically evaluate the cardiovascular consequences of multiple electrolytes across both haemodialysis and peritoneal dialysis, allowing identification of modality-specific and modality-independent risk patterns. The analytic approach combined rigorous study selection with transparent and reproducible data processing, including consistent transformation of effect estimates and careful handling of multiple exposure definitions within studies. Sensitivity analyses confirmed the stability of key findings, particularly for phosphate and magnesium, and the inclusion of both categorical and continuous models provided complementary clinical insights. However, certain limitations must be acknowledged. First, although we focused on cardiovascular mortality and SCD, many included studies did not disaggregate sudden death from broader cardiovascular death, which may obscure arrhythmic vs. non-arrhythmic mechanisms. Second limitation relates to the timing of electrolyte assessment in relation to cardiovascular death and SCD. The vast majority of included cohorts reported only baseline or time-averaged serum electrolyte levels, with follow-up extending over several years. As a result, the electrolyte values we analysed represent long-term risk markers rather than measurements obtained immediately before the fatal event. We were unable to examine short-term fluctuations, intra-individual variability, or peridialytic shifts in electrolytes that might directly precipitate arrhythmic death. This temporal separation means that our findings should be interpreted as showing associations between chronic electrolyte profiles and subsequent cardiovascular mortality/SCD, rather than identifying the exact electrolyte levels present at the time of death. Third, despite our rigorous criteria, residual confounding remains possible in observational cohorts. In particular nutritional status, volume overload, systemic inflammation, medication effects, underlying cardiac disease, dialysate composition (e.g., potassium, magnesium, sodium and calcium baths) and clinician prescription patterns (phosphate binders, magnesium supplementation, ultrafiltration targets, session length) are imperfectly captured and may influence both electrolyte levels and cardiovascular outcomes, making it difficult to disentangle whether electrolytes act as causal mediators or as integrated markers of broader illness severity. Fourth, heterogeneity was high for several electrolyte analyses (especially magnesium and potassium in PD), limiting the certainty of pooled estimates. Fifth, as always in meta-analyses of observational data, publication bias and selective reporting may distort effect estimates. Data for calcium and chloride-related markers were sparse, precluding pooled analyses for some electrolytes. Finally, although categorical analyses were generally robust, continuous models for magnesium and phosphate were highly heterogeneous due to differences in measurement units and exposure scaling, restricting their interpretability.

## 6. Conclusions

In this systematic review and meta-analysis of haemodialysis and peritoneal dialysis populations, abnormalities in several serum electrolytes were consistently associated with cardiovascular mortality, and likely with sudden cardiac death. Elevated phosphate levels were associated with higher cardiovascular mortality across modalities, and extreme potassium abnormalities conferred substantial excess risk, particularly in peritoneal dialysis. Categorical hypomagnesaemia also showed a robust association with higher cardiovascular risk, although the small number of contributing cohorts, inconsistent exposure definitions and heterogeneous continuous models mean that clinically actionable thresholds for magnesium cannot yet be defined. By contrast, the available data on calcium were sparse and inconsistent and do not support firm conclusions about its independent prognostic value. Lower serum sodium and an unfavourable Na/Cl ratio were also associated with higher cardiovascular mortality. These findings support the importance of individualized electrolyte monitoring and modality-tailored management of phosphate, magnesium, potassium and sodium as a core component of cardiovascular and sudden death risk reduction in dialysis care. Because all available data are observational, there is a need for further high-quality interventional and mechanistic studies to determine whether correcting specific electrolyte perturbations can causally reduce cardiovascular and sudden death events.

## Figures and Tables

**Table 1:** Categorical, continuous and time-varying associations between baseline serum electrolyte abnormalities and cardiovascular mortality in patients receiving peritoneal dialysis.

Electrolyte	Contrasts	*n*	HR (95% CI)	*p*	I^2^ (%)	*p* for Heterogeneity
K	Severe potassium abnormality vs. mid-range reference	5	2.05 (1.32–3.20)	0.002	>80	NR
Mg **	Low vs. normal serum magnesium	2	1.73 (1.23–2.44)	0.002	0	0.45
P	High phosphate vs. mid-range reference	4	2.03 (1.20–3.43)	0.008	≈79	–
Ca × P	High vs. low calcium × phosphate (Ca × P) product	1	2.17 (1.45–3.26)	<0.001	0 *	–
Cl	Chloride <100 mmol/L vs. >103 mmol/L	1	≈2.86 (CI NR)	NR	0 *	–
Na/Cl	Highest quartile (>1.42) vs. lowest (<1.33)	1	0.38 (0.22–0.67)	NR	0 *	–
Na	137–<139 mEq/L vs. ≥139 mEq/L	1	1.37 (0.64–2.92)	NR	0 *	–
Mg ^#^	Continuous magnesium	2	0.14 (0.00–5.36)	0.29	94	<0.001
P	Per unit increase (linear model)	2	1.01 (0.62–1.62)	0.98	NR	NR
Na/Cl	Per 0.1 unit higher Na/Cl	1	0.60 (0.46–0.78)	NR	0 *	–
Na	Per 1 mEq/L higher	1	0.95 (0.84–1.08)	NR	0 *	–

Abbreviations: NR, not reported in text; CI, confidence interval. * I^2^ = 0% by definition when k = 1; ** τ^2^ = 0.000; Q = 0.57 (df = 1), ^#^ τ^2^ = 6.50; Q = 17.59 (df = 1).

**Table 2:** Categorical, continuous and time-varying associations between baseline serum electrolyte abnormalities and cardiovascular mortality in patients receiving haemodialysis.

Electrolyte	Contrast	*n*	HR (95% CI)	*p*	I^2^ (%)	*p* for Heterogeneity
Mg **	Low vs. normal serum magnesium	3	2.17 (1.17–4.00)	0.011	26	0.26
P ^#^	High phosphate vs. reference	3	1.98 (1.36–2.68)	<0.001	79.5	0.008
K ^$^	Extreme potassium levels vs. mid-range reference	2	1.13 (0.68–1.88)	0.63	29.8	0.23
Na ^&^	Per 1 unit higher sodium	3	0.94 (0.91–0.96)	<0.001	0	0.68

Abbreviations: CI, confidence interval. ** τ^2^ = 0.085; Q = 2.72 (df = 2); ^#^ τ^2^ = 0.085; Q = 2.72 (df = 2), ^$^ τ^2^ = 0.065; ^&^ τ^2^ = 0.000; Q = 0.76 (df = 2).

**Table 3:** Categorical, continuous and time-varying associations between baseline serum electrolyte abnormalities and cardiovascular mortality in all dialysis patients combined (peritoneal dialysis and haemodialysis).

Electrolyte	Contrast	*n*	HR (95% CI)	*p*	I^2^ (%)
P *	High phosphate vs. reference	6/7	2.09 (1.60–2.72)	<0.001	≈79
Mg	Low vs. normal magnesium	5	1.82 (1.39–2.38)	<0.001	0
K	Extreme potassium abnormality vs. mid-range	7	1.73 (1.22–2.46)	0.002	≈81
Mg	Per unit higher magnesium		0.68 (0.50–0.93)	0.015	≈82
Na	Per 1 unit higher sodium	4	0.94 (0.92–0.96)	<0.001	0

Abbreviations: CI, confidence interval; * τ^2^ = 0.085.

## Data Availability

The original contributions presented in this study are included in the article/Supplementary Material. Further inquiries can be directed to the corresponding author.

## Supplementary Materials

The following supporting information can be downloaded at: https://www.mdpi.com/article/10.3390/biomedicines14030605/s1. Supplementary Table S1: PRISMA Search Strategy; Supplementary Table S2: Risk of bias; Supplementary Table S3: Certainty of evidence (GRADE).
