# Supplementary material for: Impact of Serum Phosphate, Potassium and Other Electrolyte Levels on Sudden Cardiac Death and Cardiovascular Mortality in Haemodialysis and Peritoneal Dialysis: A Systematic Review and Meta-Analysis
Source: Biomedicines. 2026 Mar 9;14(3):605. doi: 10.3390/biomedicines14030605 (PMC13024601; doi:10.3390/biomedicines14030605)
Supplement: Supplementary file 1 [file biomedicines-14-00605-s001.zip › biomedicines-4035392-supplementary.pdf]

Supplementary Table S1. PRISMA Search Strategy

Database	Date of search	Search strategy	Search period	Results (n)
PubMed	[1.10.2025]	(("sudden cardiac death"[MeSH Terms] OR "sudden cardiac death"[Title/Abstract] OR "SCD"[Title/Abstract] OR "cardiovascular death"[Title/Abstract] OR "cardiovascular mortality"[Title/Abstract]) AND ("Electrolyte Imbalance"[MeSH Terms] OR "serum electrolyte imbalance"[Title/Abstract] OR "serum potassium"[Title/Abstract] OR "serum sodium"[Title/Abstract] OR "serum calcium"[Title/Abstract] OR "serum magnesium"[Title/Abstract]) AND ("Renal Dialysis"[MeSH Terms] OR "dialysis"[Title/Abstract] OR "hemodialysis"[Title/Abstract] OR "haemodialysis"[Title/Abstract] OR "peritoneal dialysis"[Title/Abstract] OR "HD"[Title/Abstract] OR "PD"[Title/Abstract] OR "CAPD"[Title/Abstract])).	2010-2025	41
Cochrane Library	[2.10.2025]	("sudden cardiac death":ti,ab,kw OR SCD:ti,ab,kw OR "cardiovascular death":ti,ab,kw OR "cardiovascular mortality":ti,ab,kw) AND ("serum potassium":ti,ab,kw OR "serum sodium":ti,ab,kw OR "serum calcium":ti,ab,kw OR "serum magnesium":ti,ab,kw OR "serum electrolyte imbalance":ti,ab,kw) AND ("hemodialysis":ti,ab,kw OR "haemodialysis":ti,ab,kw OR "peritoneal dialysis":ti,ab,kw OR dialysis:ti,ab,kw OR HD:ti,ab,kw OR PD:ti,ab,kw OR CAPD:ti,ab,kw)	2010-2025	[36 (12 reviews, 24 trials)]
Google Scholar	[5.10.2025]	hemodialysis" OR "peritoneal dialysis" AND "serum potassium" OR "serum sodium" OR "serum calcium" OR "serum magnesium" AND "sudden cardiac death" OR "cardio-vascular mortality" OR "cardiovascular death".	2010-2025	10,500 hits, 200 studies screened

SUPPLEMENTARY TABLE S2. Risk of bias

Publication	RoB_Confounding	RoB Selection	RoB Exposure	RoB Deviations	RoB Missing Data	RoB Outcome Measurement	RoB Reporting	RoB Overall
Cai_2016.pdf	Moderate	Moderate	Moderate	Low	Moderate	Moderate	Moderate	Moderate
Chang_2014.pdf	Moderate	Moderate	Moderate	Low	Moderate	Moderate	Moderate	Moderate
Eriguchi_2019_V.pdf	Moderate	Moderate	Moderate	Low	Moderate	Moderate	Moderate	Moderate
Fujisaki_2021.pdf	Moderate	Moderate	Moderate	Low	Moderate	Moderate	Moderate	Moderate
Goto_2024.pdf	Moderate	Moderate	Moderate	Low	Moderate	Moderate	Moderate	Moderate
Hecking_2012.pdf	Moderate	Moderate	Moderate	Low	Moderate	Moderate	Moderate	Moderate
Huang_2021.pdf	Moderate	Moderate	Moderate	Low	Moderate	Moderate	Moderate	Moderate
Huang_2023.pdf	Moderate	Moderate	Moderate	Low	Moderate	Moderate	Moderate	Moderate
Kurita_2015.pdf	Moderate	Moderate	Moderate	Low	Moderate	Moderate	Moderate	Moderate
Li_2015b.pdf	Moderate	Moderate	Moderate	Low	Moderate	Moderate	Moderate	Moderate
Li_2021_V.pdf	Moderate	Moderate	Moderate	Low	Moderate	Moderate	Moderate	Moderate
Li_2022_V.pdf	Moderate	Moderate	Moderate	Low	Moderate	Moderate	Moderate	Moderate
Lu_2020.pdf	Moderate	Moderate	Moderate	Low	Moderate	Moderate	Moderate	Moderate
Matias_2014.pdf	Moderate	Moderate	Moderate	Low	Moderate	Moderate	Moderate	Moderate
Men_2024.pdf	Moderate	Moderate	Moderate	Low	Moderate	Moderate	Moderate	Moderate
Mizuiri_2019.pdf	Moderate	Moderate	Moderate	Low	Moderate	Moderate	Moderate	Moderate
Petrakis_2024.pdf	Moderate	Moderate	Moderate	Low	Moderate	Moderate	Moderate	Moderate
Pun_2011.pdf	Moderate	Moderate	Moderate	Low	Moderate	Low	Moderate	Moderate

Pun_2013.pdf	Moderate	Moderate	Moderate	Low	Moderate	Low	Moderate	Moderate
Ribeiro_2015.pdf	Moderate	Moderate	Moderate	Low	Moderate	Moderate	Moderate	Moderate
Sakaguchi_2014.pdf	Moderate	Moderate	Moderate	Low	Moderate	Moderate	Moderate	Moderate
Sato_2018.pdf	Moderate	Moderate	Moderate	Low	Moderate	Moderate	Moderate	Moderate
Tiong_2021_V.pdf	Moderate	Moderate	Moderate	Low	Moderate	Moderate	Moderate	Moderate
Torlen_2012_V.pdf	Moderate	Moderate	Moderate	Low	Moderate	Moderate	Moderate	Moderate
Truyts_2021.pdf	Moderate	Moderate	Moderate	Low	Moderate	Moderate	Moderate	Moderate
Waikar_2011.pdf	Moderate	Moderate	Moderate	Low	Moderate	Moderate	Moderate	Moderate
Wu_2019.pdf	Moderate	Moderate	Moderate	Low	Moderate	Moderate	Moderate	Moderate
Xu_2014_V.pdf	Moderate	Moderate	Moderate	Low	Moderate	Moderate	Moderate	Moderate
Ye_2018.pdf	Moderate	Moderate	Moderate	Low	Moderate	Moderate	Moderate	Moderate
You_2024.pdf	Moderate	Moderate	Moderate	Low	Moderate	Moderate	Moderate	Moderate
Yusuf_2016.pdf	Moderate	Moderate	Moderate	Low	Moderate	Moderate	Moderate	Moderate
Zhang_2021_V.pdf	Moderate	Moderate	Moderate	Low	Moderate	Moderate	Moderate	Moderate
Zhou_2021.pdf	Moderate	Moderate	Moderate	Low	Moderate	Moderate	Moderate	Moderate
de Roij_2015.pdf	Moderate	Moderate	Moderate	Low	Moderate	Moderate	Moderate	Moderate
wu2019b.pdf	Moderate	Moderate	Moderate	Low	Moderate	Moderate	Moderate	Moderate

SUPPLEMENTARY TABLE S3. Certainity of evidence (GRADE)

K categorical in PD	low
Mg categorical in PD	very low
P categorical in PD	low

CaxP categorical in PD	very low
Cl categorical in PD	very low
Na/Cl categorical in PD	very low
Na categorical in PD	very low
Mg continuous in PD	very low
P continuous in PD	very low
Na/Cl continuous in PD	very low
Na continuous in PD	very low
K categorical in HD	very low
Mg categorical in HD	very low
P categorical in HD	very low
Na continuous in HD	very low
P categorical in HD+PD	low
Mg categorical in HD+PD	low
K categorical in HD+PD	low
Mg continuous in HD+PD	very low
Na continuous in HD+PD	very low
